# Association between hydroxychloroquine use and risk of diabetes mellitus in systemic lupus erythematosus and rheumatoid arthritis: a UK Biobank-based study

**DOI:** 10.3389/fendo.2024.1381321

**Published:** 2024-11-06

**Authors:** Chen-Xia Li, Meng-Lin Fan, Bo-Wen Pang, Xing-Jian Zhou, Hong-Zi Zhang, Jing-Jing Zeng, Shao-yong Xu, Jin-Kui Yang

**Affiliations:** ^1^ Department of Endocrinology, Beijing Tongren Hospital, Capital Medical University, Beijing, China; ^2^ Department of Endocrinology, Xiangyang No. 1 People’s Hospital, Hubei University of Medicine, Xiangyang, Hubei, China; ^3^ Department of Endocrinology, Xiangyang Central Hospital, Affiliated Hospital of Hubei University of Arts and Science, Xiangyang, Hubei, China; ^4^ Center for Clinical Evidence-Based and Translational Medicine, Xiangyang Central Hospital, Affiliated Hospital of Hubei University of Arts and Science, Xiangyang, Hubei, China; ^5^ Department of Rheumatology and Immunology, Xiangyang No. 1 People’s Hospital, Hubei University of Medicine, Xiangyang, Hubei, China

**Keywords:** hydroxychloroquine, diabetes mellitus, UK Biobank, risk, prospective cohort study, rheumatic disease

## Abstract

**Context/Objectives:**

Hydroxychoroquine has hypoglycemic effects and may reduce the risk of diabetes mellitus (DM). We determined the association between hydroxychoroquine use and the incidence of DM in a population-based cohort of pations with Rheumatic disease

**Methods:**

A prospective cohort study among 502392 Potentially eligible participants in the context of UK Biobank, recruitment to the database began between 2006 and 2010. Patients diagnosed with diabetes and fasting glucose greater than or equal to 7 mmol/L at baseline (n=619) were excluded and patients diagnosed with either RA or SLE at baseline (n=6793) were followed up until 2022. Diagnosis was recorded using the International Classification of Diseases, tenth edition (ICD-10) code. The mean follow-up was 13.78 years and the primary outcome was newly recorded type 2 diabetes mellitus (T2DM), with the time of onset of diabetes as the follow-up endpoint date.

**Results:**

During a median follow-up period of 13.78 (12.93, 14.49) years, diabetes developed in 537 participants, with an incidence of 7.9%. New diabetes cases not taking hydroxychloroquine and taking hydroxychloroquine was 504 (8.03%) and 33 (6.36%), respectively. In univariate models, the hazard ratio for diabetes was 0.89 (95% confidence interval, 0.81-0.98, *P*=0.014) for hydroxychloroquine users compared with those not taking hydroxychloroquine. After adjusting for age, sex, race, education level, and BMI the hazard ratio for incident diabetes among hydroxychloroquine users was 0.88 (95% confidence interval, 0.80-0.97, *P*=0.008). In complete multivariate model hazard ratio for hydroxychloroquine was 0.87 (95% confidence interval, 0.79- 0.96, *P*=0.005).

**Conclusion:**

Hydroxychloroquine was associated with decreased risk of DM among rheumatoid arthritis patients, our data taken together with correlational studies, warrant further investigation of the potential preventive effect of hydroxychloroquine against T2DM.

## Introduction

Rheumatic disease or rheumatism is a type of autoimmune inflammation that involves multiple organs and tissues of the entire body (1, 2), and it is a significant cause of disability and decline in the daily quality of life (3–5). The mortality rate for patients with rheumatic disease is 1.5 times higher than that for the general population (6), with the principal representative diseases being systemic lupus erythematosus (SLE) and rheumatoid arthritis (RA), at prevalence rates of 0.03% and 0.28% (7), respectively. Epidemiologic studies also describe an increased risk of diabetes in individuals with rheumatism, which may be related to multiple factors such as disease-related inflammation, premature menopause, forced inactivity, and drugs used to treat the disease (such as glucocorticoids and immunosuppressants) (5, 8). Considering that diabetes is also a recognized risk factor for cardiovascular death, reducing the risk of developing diabetes in those with rheumatism is crucial (9, 10).

Hydroxychloroquine (HCQ) is a classic traditional synthetic anti-rheumatic drug (2, 11). HCQ has become a background drug for the treatment of SLE (12, 13). ACR guidelines conditionally recommend the use of HCQ in the initial treatment of patients with mild to moderate RA (14). Cases of reduced blood sugar levels after hydroxychloroquine treatment were first reported in 1984. In addition, studies have found that hydroxychloroquine can reduce cardiovascular risk by controlling blood sugar levels when used to treat rheumatoid arthritis and systemic lupus erythematosus (15). Furthermore, HCQ is a known ion channel inhibitor (16), but this property has not been linked to its effect on blood sugar.

The relationship between hydroxychloroquine and diabetes mellitus (DM) has been a cause of increasing concern (17, 18). Hydroxychloroquine, as inferred from chloroquine studies, improves glucose homeostasis and reduces diabetic incidence (19, 20). Unfortunately, studies that have focused on the efficacy of hydroxychloroquine on blood glucose and diabetes risk in patients with rheumatism have proven inadequate, and there are only a few clinical studies in the extant literature. Therefore, it is particularly important to conduct larger population studies with long follow-up times. Based on these considerations, we analyzed the UK Biobank to explore whether hydroxychloroquine use was associated with a lower risk of diabetes in patients with rheumatism.

## Materials and methods

### Study design and participants

**Figure 1:**
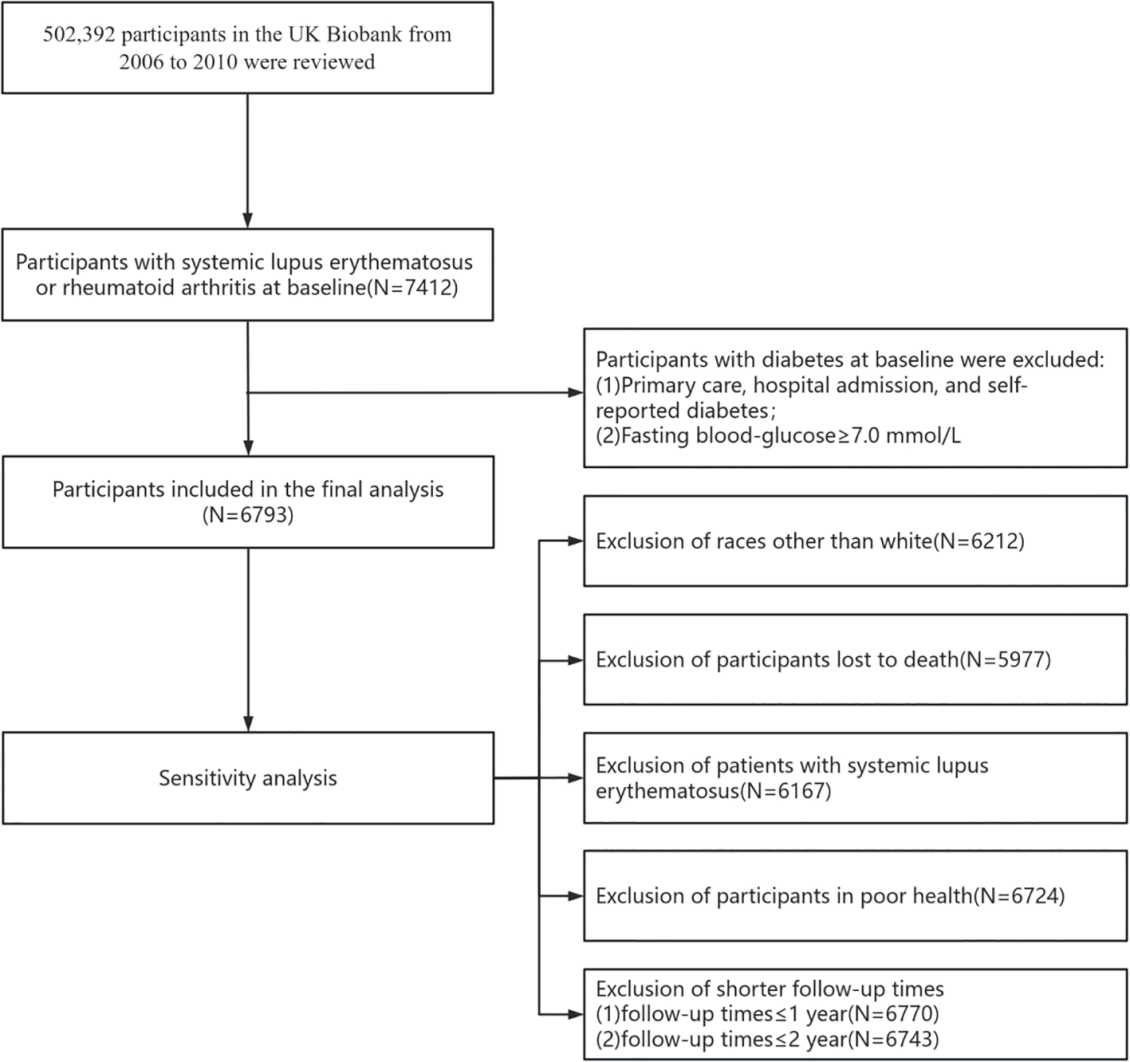
Participant flowchart.

The UK Biobank is a large population-based, prospective cohort study that comprises more than one-half million participants aged 40-69 years recruited in the UK between 2006 and 2010. Each participant underwent a touch-screen questionnaire, oral interviews, and body measurements and provided a biological sample. The UK Biobank has received ethical approval from the North West Multi-centre Research Ethics Committee (MREC), and all participants have provided informed written consent. Participants with RA or SLE prior to the baseline survey were included in our study (N=7412), and participants with a baseline diagnosis of diabetes or a fasting blood glucose (FBG) of ≥7 mmol/L were excluded (N=619), resulting in 6793 participants (**Figure 1**).

### Exposure and outcomes assessment

Hydroxychloroquine was administered orally by trained nurses. This variable included weekly and monthly medication data for routine treatment, excluding short-term medication.

Participant health status was collected using records that contained primary healthcare data, hospital admission data, and self-reported medical status at evaluation centers. Diagnoses were recorded using the International Classification of Diseases, 10^th^ edition (ICD-10), and patients with RA (M05, M06) and SLE (M32) were identified. The primary outcome of our study was the risk of developing T2DM (E11). Participants were accepted for follow-up until diagnosis of T2DM, death, loss to follow-up, or at the date of available data (September 30, 2021), whichever was earlier.

### Covariates

Possible confounding factors included sociodemographic levels, lifestyle, a family history of diabetes, and baseline FBG. Information on age, sex, Ethnicity, education level, physical activity, smoking, alcohol consumption, dietary habits, and a family history of diabetes was collected by baseline questionnaires in the UK Biobank. In this study, ethnicity was classified as White, Asian or Asian British, Black or Black British and other ethnic group. Education level was categorized as college or university degree, A/AS level or equivalent, O levels/GCSEs or equivalent and others. Smoking and alcohol consumption were both categorized as never, previous and current. Regarding diet, participants were asked about their intake of sugary beverages, intake of vegetables and fruits, and frequency of their consumption of fish, red meat, and processed meat. In the study, diet frequency was categorized as less than once a week, once per week and more than once per week. Body measurements were performed by trained nurses who applied a uniform method to collect participant height and weight, and body mass index (BMI) was calculated as BMI (kg/m^2^) = weight (kg)/height (m)^2^. We measured baseline FBG using hexokinase and a Beckman Coulter AU5800 (Beckman, USA).

### Statistical analysis

For descriptive analyses of baseline characteristics, categorical variables are expressed as frequencies (percentages) and continuous variables as means ± standard deviations, or medians (interquartile ranges). Baseline characteristics were compared with or without hydroxychloroquine using Chi-squared tests, one-way analysis of variance (parametric), or the Kruskal-wallis test (nonparametric) followed by Tukey’s test, and *P*<0.05 indicated a statistical significance.

We exploited Cox proportional hazards models to analyze the association between hydroxychloroquine use and diabetes risk in participants with RA, SLE and Rheumatic Disease (RA or SLE), respectively. Schoenfeld residuals validated the assumption of equal proportional risk (*P*>0.05). The time variable was follow-up time from baseline (2006-2010) to diabetes onset or follow-up cut-off date (2021). Results are reported as hazard ratio (HR) and 95% confidence interval (CI). In model 2, we further adjusted for smoking, alcohol consumption, physical activity, and diet (including intake of sugar or sugar-sweetened beverages, vegetables, fruits, processed meats, red meat, and oily fish). In model 3, we further adjusted for family history of diabetes and FBG.

We subsequently performed several additional analyses to assess the robustness of the results. We first examined by stratified analysis whether the association between hydroxychloroquine and diabetes risk varied by type of rheumatic immune disease (RA or SLE), age (≤60 years vs. >60 years), BMI, sex, or FBG. The association between hydroxychloroquine and diabetes risk was also investigated by a series of sensitivity analyses. We initially excluded participants with SLE, included only RA patients for analysis, and performed subgroup analyses for RA patients. Second, the association between hydroxychloroquine and diabetes risk was only evaluated in individuals who were white, thus excluding other races. Third, we excluded participants who developed diabetes within 1 or 2 years of follow-up. Fourth, eliminating participants who self-reported poor health reduced the impact of poor health on lifestyle behavior. Fifth, we excluded participants who died before the endpoint event. We performed all statistical analyses using SAS 9.4 software.

## Results

### Population characteristics

Baseline characteristics of the study population are shown in **Table 1**: 6793 participants with SLE or RA were included in the study for analysis, with a median age of 60.00 (54.00, 65.00) years, of whom 4852 (71.43%) were female and 6085 (89.58%) had RA. Compared with those who did not take hydroxychloroquine, participants who took hydroxychloroquine were more likely to be female, younger, more educated, nonwhite, former smokers and drinkers; with less physical activity, greater fruit intake, processed meat intake, and lower baseline FBG (*P*<0.05).

**Table 1:** Baseline population characteristics.

	No hydroxychloroquine (N = 6274)	Hydroxychloroquine (N = 519)	Overall (N = 6793)	*P*
**Female, n (%)**	4425 (70.53)	427 (82.27)	4852 (71.43)	<0.001
**Age**	60.00 (54.00, 65.00)	59 (52.00, 64.00)	60.00 (54.00, 65.00)	<0.001
**Ethnicity**				0.001
White	5757 (91.76)	455 (87.67)	6212 (91.45)	
Asian or Asian British	144 (2.30)	13 (2.50)	157 (2.31)	
Black or Black British	103 (1.64)	19 (3.66)	122 (1.80)	
Other ethnic group	270 (4.30)	32 (6.17)	302 (4.45)	
**Education**				0.003
College or university degree	1379 (21.98)	139 (26.78)	1518 (22.35)	
A/AS level or equivalent	628 (10.01)	66 (12.72)	694 (10.22)	
O levels/GCSEs or equivalent	1620 (25.82)	132 (25.43)	1752 (25.79)	
Other	2647 (42.19)	182 (35.07)	2829 (41.65)	
**BMI (kg/m^2^)**	27.29 (24.37, 30.78)	26.95(23.85, 30.99)	27.82 (24.32, 30.79)	0.257
**Smoking**				0.024
Never	3017 (48.50)	253 (48.94)	3270 (48.54)	
Previous	2404 (38.65)	218 (42.17)	2622 (38.92)	
Current	799 (12.85)	46 (8.90)	845 (12.54)	
**Alcohol consumption**				0.499
Never	451 (7.21)	39 (7.51)	490 (7.23)	
Previous	458 (7.32)	45 (8.67)	503 (7.42)	
Current	5347 (85.47)	435 (83.82)	5782 (85.34)	
**Physical activity (minutes/week)**	2372.86 (810.00, 2472.00)	1633.00 (642.00, 2372.86)	2372.86 (792.00, 2400.00)	<0.001
**Vegetables (tablespoons/day)**	5.00 (3.00, 6.00)	5.00 (3.00, 6.00)	5.00 (3.00, 6.00)	0.142
**Fruit (pieces/day)**	3.00 (2.00, 4.00)	3.00 (2.00, 4.00)	3.00 (2.00, 4.00)	0.020
**Sugared beverages**				0.326
No	1286 (20.50)	97 (18.69)	1383 (20.36)	
Yes	4988 (79.50)	422 (81.31)	5410 (79.64)	
**Oily fish**				0.063
Less than once per week	2755 (44.36)	238 (46.12)	2993 (44.49)	
Once per week	2243 (36.11)	199 (38.57)	2442 (36.30)	
More than once per week	1213 (19.53)	79 (15.31)	1292 (19.21)	
**Processed meat**				0.009
Less than once per week	2671 (42.87)	250 (48.36)	2921 (43.29)	
Once per week	1818 (29.18)	153 (29.59)	1971 (29.21)	
More than once per week	1741 (27.95)	114 (22.05)	1855 (27.49)	
**Beef**				0.230
Less than once per week	3513 (56.46)	312 (60.35)	3825 (56.76)	
Once per week	1923 (30.91)	146 (28.24)	2069 (30.70)	
More than once per week	786 (12.63)	59 (11.41)	845 (12.54)	
**Mutton**				0.143
Less than once per week	4529 (72.92)	392 (76.12)	4921 (73.16)	
Once per week	1462 (23.54)	102 (19.81)	1564 (23.25)	
More than once per week	220 (3.54)	21 (4.08)	241 (3.58)	
**Pork**				0.918
Less than once per week	4600 (74.12)	384 (74.71)	4984 (74.17)	
Once per week	1381 (22.25)	113 (21.98)	1494 (22.23)	
More than once per week	225 (3.63)	17 (3.31)	242 (3.60)	
**FBG (mmol/L)**	4.97 (4.65, 5.22)	4.95 (4.57, 5.16)	4.97 (4.64, 5.21)	0.022
**Family history of diabetes**	1432 (22.82)	105 (20.23)	1537 (22.63)	0.175
**Immune rheumatic disease**				<0.001
Systemic lupus erythematosus	490 (7.81)	136 (26.20)	626 (9.22)	
Rheumatoid arthritis	5734 (91.39)	351 (67.63)	6085 (89.58)	
Afflicted by both diseases	50 (0.80)	32 (6.17)	82 (1.21)	

### Relationship between Hydroxychloroquine and diabetes mellitus

During a median follow-up period of 13.78 (12.93, 14.49) years, diabetes developed in 537 participants, with an incidence of 7.9%. The numbers of individuals not taking hydroxychloroquine vs. taking hydroxychloroquine were 504 (8.03%) and 33 (6.36%), respectively.

**Table 2** shows the association between hydroxychloroquine use and diabetes risk. Participants with RA taking hydroxychloroquine had reduced risk of diabetes compared with those not taking hydroxychloroquine (HR=0.82 [95%CI: 0.73-0.92], *P*< 0.001). However, in the SLE population, hydroxychloroquine use is not significantly associated with the risk of diabetes mellitus (HR=1.07 [95%CI: 0.86-1.31], *P*= 0.553). In participants with rheumatic immune, using univariate models, the hazard ratio for diabetes was 0.89 (95% CI: 0.81–0.98) for hydroxychloroquine users when compared with those not taking hydroxychloroquine (*P*=0.014). The hazard ratio for DM after adjusting for age, sex, race, educational level, and BMI (model 1) was 0.88 (95% CI: 0.80-0.97) for hydroxychloroquine (*P*=0.008), and the hazard ratio for hydroxychloroquine was 0.87 (95% CI: 0.79–0.96) in model 3 (*P*=0.005) (see **Supplementary Table 1** for details of the variable adjustments).

**Table 2:** Association between hydroxychloroquine use and diabetes incidence in rheumatic immune patients.

	No hydroxychloroquine	Hydroxychloroquine	*P*
Participants with rheumatoid arthritis			
Number of event	473	22	
Incidence	5734	351	
Number of observers	8.25	6.27	0.188
Model 0	1.00 (Reference)	0.82(0.73,0.92)	<0.001
Model 1	1.00 (Reference)	0.82(0.73,0.91)	<0.001
Model 2	1.00 (Reference)	0.81(0.73,0.91)	<0.001
Model 3	1.00 (Reference)	0.82 (0.73, 0.92)	<0.001
Participants with systemic lupus erythematosus patients			
Number of event	27	136	
Number of observers	490	8	
Incidence	5.51	6.27	0.867
Model 0	1.00 (Reference)	1.02(0.84,1.25)	0.830
Model 1	1.00 (Reference)	1.02(0.83,1.25)	0.841
Model 2	1.00 (Reference)	1.07(0.87.1.31)	0.555
Model 3	1.00 (Reference)	1.07 (0.86, 1.31)	0.553
Participants with rheumatic immune patients			
Number of event	504	33	
Number of observers	6274	519	
Incidence	8.03	6.36	0.174
Model 0	1.00 (Reference)	0.89 (0.81, 0.98)	0.014
Model 1	1.00 (Reference)	0.88 (0.80, 0.97)	0.008
Model 2	1.00 (Reference)	0.87 (0.79, 0.95)	0.003
Model 3	1.00 (Reference)	0.87 (0.79, 0.96)	0.005

BMI, body mass index; FBG, baseline fasting blood glucose.

Model 0 was analyzed with single-factor analysis.

Model 1 was adjusted for age, sex, ethnicity, educational level, and BMI.

Model 2 was adjusted for age, sex, ethnicity, educational level, BMI, smoking, alcohol consumption, physical activity, and diet (including intake of sugar or sugar-sweetened beverages, vegetables, fruits, processed meats, red meat, and oily fish).

Model 3 was adjusted for age, sex, ethnicity, educational level, BMI, smoking, alcohol consumption, physical activity, diet, family history of diabetes, and FBG.

### Subgroup analyses

**Figure 2:**
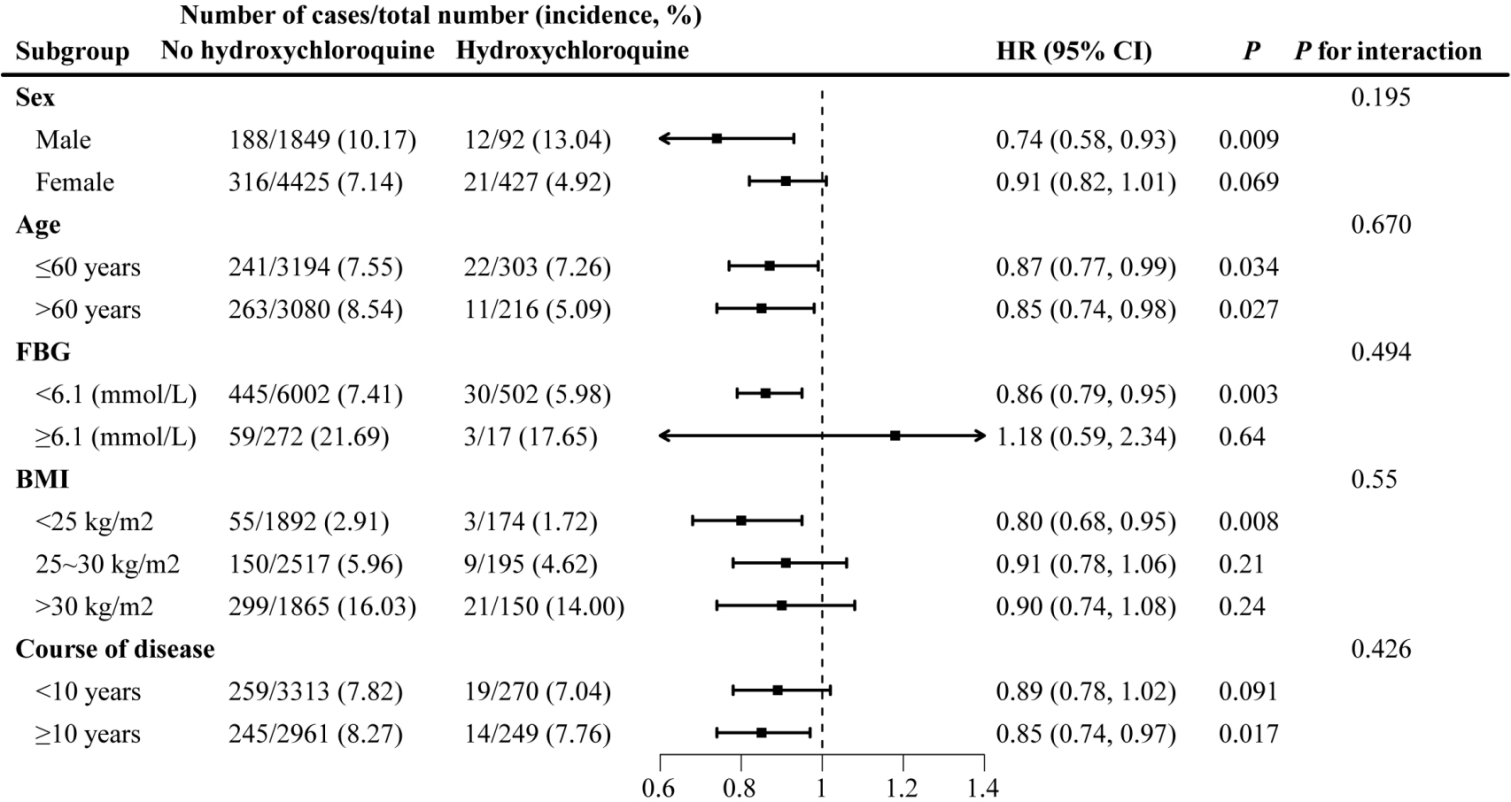
Subgroup analysis: stratified analysis by sex, age, baseline FBG, BMI and course of disease.

Subgroup analyses showed consistent results as stratified by age, with relative risks of diabetes for participants aged 60 years or older/younger of 0.87 (95% CI: 0.77–0.99, *P*=0.034) and 0.85 (95% CI: 0.74–0.98, *P*=0.027) for hydroxychloroquine users compared with non-users. Hydroxychloroquine significantly reduced the risk of diabetes among male participants with low baseline FBG (<6.1 mmol/L), non-overweight obesity, longer duration of rheumatic immune disease (≥10 years), and RA (*P*<0.05) (**Figure 2**). The association of hydroxychloroquine with diabetes was not significantly different in all subgroups (*P* for interaction >0.05).

### Sensitivity analysis

In sensitivity analyses that excluded SLE, race other than white, poor health within one or two years of follow-up, lost to follow-up due to death, and hydroxychloroquine use were associated with a similar reduction in diabetes risk (*P*=0.002) (**Table 3**). We observed no association between hydroxychloroquine use and diabetes onset among obese participants with a baseline FBG ≥6.1 mmol/L (*P*>0.05).

**Table 3:** Sensitivity analyses.

	Number of cases/total number (incidence, %)		HR (95% CI)		*P*
	No hydroxychloroquine	Hydroxychloroquine	No hydroxychloroquine	Hydroxychloroquine	
Eliminating systemic lupus erythematosus	477/5784 (8.52)	25/383 (6.53)	1.00 (Reference)	0.84 (0.75, 0.94)	0.002
Ethnicity: White	433/575 (7.52)	26/455 (5.71)	1.00 (Reference)	0.84 (0.76, 0.93)	<0.001
Excluding too short a follow-up					
≤1 year	484/6254 (7.74)	30/516 (7.62)	1.00 (Reference)	0.87 (0.79, 0.96)	0.005
≤2 years	458/6228 (7.35)	29/515 (7.62)	1.00 (Reference)	0.87 (0.79, 0.96)	0.005
Eliminating poor health	496/6209 (8.66)	33/515 (5.88)	1.00 (Reference)	0.87 (0.80, 0.96)	0.005
Excluding loss to follow-up due to death	504/5534 (9.11)	33/443 (7.45)	1.00 (Reference)	0.90 (0.81, 1.00)	0.041

(1) Excluding other races, including only whites. (2) Excluding patients with systemic lupus erythematosus, including patients with rheumatoid arthritis. (3) Excluding participants with shorter follow-up. (4) Excluding patients who self-reported poor health. (5) Excluding participants who died before diabetes onset.

## Discussion

We herein analyzed the association between hydroxychloroquine and diabetes in a UK population sample of 500,000 and found that hydroxychloroquine use reduced the risk of diabetes by a 13% (HR=0.87 [95%CI: 0.79-0.96], *P*=0.005), particularly in RA patients. We also conducted a large number of sensitivity analyses, with consistent results. The advantages of the present study were our large population cohort, adequate subgroup analyses, and sensitivity analyses.

Previous investigators have demonstrated that hydroxychloroquine reduced the risk of diabetes in rheumatic diseases, and this was consistent with our findings. For example, the results of a prospective, multicenter observational study (21) showed that patients with RA who took hydroxychloroquine reflected a hazard ratio of 0.62 (95% CI, 0.42–0.92) with respect to developing diabetes compared with non-users. Ozen and colleagues conducted another retrospective cohort study that encompassed 13,669 patients with RA and depicted an adjusted hazard ratio of 0.67 (95% CI, 0.77–0.80) (22). Patients with RA who received hydroxychloroquine thus generated a significantly reduced risk of diabetes compared with those who did not. Many studies have consistently revealed that hydroxychloroquine use reduced the risk of type 2 diabetes in RA patients (20, 22–26), but there are few extant studies on hydroxychloroquine in SLE patients. Salmasi et al (27) ascertained that the use of antimalarial drugs can effectively reduce the risk of type 2 diabetes in SLE patients by continuously following 1498 SLE patients for 4.62 years. Their findings differed from our study in which we did not uncover an association between hydroxychloroquine and type 2 diabetes risk in SLE patients, but this difference might be explained by the limitations of the present study. First, we had a very small number of SLE subjects who used hydroxychloroquine in the current study, with a correspondingly small number of diabetes endpoint events: only 136 SLE patients used hydroxychloroquine, of whom only eight developed DM, implying that this study may not have reflected enough power for us to discern a modest association between the two conditions.

In addition, our data lacked specific information on patients’ actual use of hydroxychloroquine. Previous studies have shown a dose-dependent protective effect of hydroxychloroquine on diabetes in SLE patients. For example, Chen et al. (7) ascertained that after 10 years of follow-up of newly diagnosed SLE patients, hydroxychloroquine was dose-dependently associated with a reduced risk of diabetes. Patients with cumulative hydroxychloroquine doses of ≥129 g exhibited the lowest hazard ratio for diabetes (HR 0.26 [95% CI: 0.18-0.37], *P*<0.001); however, patients with cumulative hydroxychloroquine doses <129 g did not avoid diabetes. Unfortunately, the UK Biobank database does not contain data on hydroxychloroquine doses used by patients, and the negative results in this study may have been due to our low drug doses. One cohort study showed that patients who persisted in using hydroxychloroquine were 39% less likely to develop T2DM compared with patients who halted treatment (27). Nam et al. in a case-control study found that the hydroxychloroquine adjusted odds ratio of 0.76 with a cumulative exposure time >270 days/year was associated with a significantly reduced risk of diabetes (25). While the specific pattern of diabetes risk reduction with hydroxychloroquine remains unclear, our study is consistent with many previous studies that depicted hydroxychloroquine as reducing the development of type 2 diabetes in rheumatic patients.

Given the aforementioned limitations, an association between hydroxychloroquine and diabetes in SLE patients cannot be eliminated, although we uncovered no significant association in the present study. Our findings rather highlight the need for further research to determine the intriguing question of whether hydroxychloroquine protects SLE patients from a lower incidence of type 2 diabetes. Randomized controlled trials with larger sample sizes are therefore needed to verify the action of hydroxychloroquine on blood glucose metabolism among patients with rheumatism. The study was also limited by confounding factors that did not account for other drugs or different treatment strategies administered by physicians. A retrospective cohort study of 12880 patients (24) demonstrated that individuals with RA or psoriasis who used hydroxychloroquine had a lower risk of diabetes than those who used other antirheumatic drugs, with a hazard ratio of 0.54 (95% CI, 0.36–0.80). An individual drug analysis of 5530 RA cohort studies revealed that hydroxychloroquine reduced the type 2 diabetes hazard ratio of 0.52 (95% CI, 0.42–0.65) (26). These findings suggested that the protective effect of hydroxychloroquine on diabetes was independent of the effect of disease treatment. The protective effect of hydroxychloroquine on blood glucose metabolism is not only observed in autoimmune diseases but also in individuals without systemic inflammation but taking hydroxychloroquine to improve insulin sensitivity. A growing body of evidence therefore supports a protective association between hydroxychloroquine and type 2 diabetes (10, 28–30), but its functional underlying mechanisms in the prevention of diabetes are not well understood.

Our study did not address the mechanism of action by which hydroxychloroquine affects blood sugar, and this warrants further investigation. The primary defects in type 2 diabetics are the dysfunction of beta-islet cells and impaired insulin secretion in response to glucose stimulation (31), which involve a series of ion-channel activities (32). Given that HCQ treatment reduces insulin requirements (15, 18, 28, 33), we hypothesize that its mechanism of action on glucose metabolism may be related to reduced insulin degradation through changes in lysosomal enzyme activity and endosomal pH (15). Professor Yang’s team (34) reported a “new switch” role of the KCNH6 channel in insulin secretion (35), the team also found that KCNH2 potassium channel enhances endogenous incretin secretion (36), and whether the characterization of hydroxychloroquine ion channel inhibitor (16) plays an important role in regulating blood glucose hormone secretion is worthy of further study. Based on these studies, we hypothesize that hydroxychloroquine regulates blood glucose levels by inhibiting potassium channels to promote insulin and/or incretin secretion.

Collectively, our data support a significant association between hydroxychloroquine treatment and a reduced risk of type 2 diabetes in patients with rheumatism. Studies have shown that hydroxychloroquine is a potentially effective drug for the prevention of type 2 diabetes, at least in patients with RA. In the future, high-quality randomized controlled trials are needed to verify the hypoglycemic effect of hydroxychloroquine, and basic studies are also needed to explore its hypoglycemic mechanism of action.

## Data Availability

The original contributions presented in the study are included in the article/**Supplementary Material**. Further inquiries can be directed to the corresponding author/s.
