# Supplementary material for: Association between hydroxychloroquine use and risk of diabetes mellitus in systemic lupus erythematosus and rheumatoid arthritis: a UK Biobank-based study
Source: Front Endocrinol (Lausanne). 2024 Nov 6;15:1381321. doi: 10.3389/fendo.2024.1381321 (PMC11576225; doi:10.3389/fendo.2024.1381321)
Supplement: Supplementary file 1 [file Table1.docx]

**Table S1.** HR (95% CI) of hydroxychloroquine intake and diabetes incidence in patients with rheumatic immune disease and model adjustment details

|  | Model 0 | |  | Model 1 | |  | Model 2 | |  | Model 3 | |
| --- | --- | --- | --- | --- | --- | --- | --- | --- | --- | --- | --- |
|  | HR (95% CI) | *P* |  | HR (95% CI) | *P* |  | HR (95% CI) | *P* |  | HR (95% CI) | *P* |
| Hydroxychloroquine |  |  |  |  |  |  |  |  |  |  |  |
|  |  |  |  |  |  |  |  |  |  |  |  |
| No | 1.00 (Reference) |  |  | 1.00 (Reference) |  |  | 1.00 (Reference) |  |  | 1.00 (Reference) |  |
| Yes | 0.89 (0.81, 0.98) | 0.014 |  | 0.88 (0.80, 0.97) | 0.008 |  | 0.87 (0.79, 0.95) | 0.003 |  | 0.87 (0.79, 0.96) | 0.005 |
| Sex |  |  |  |  |  |  |  |  |  |  |  |
| Male | - | - |  | 1.00 (Reference) |  |  | 1.00 (Reference) |  |  | 1.00 (Reference) |  |
| Female | - | - |  | 0.99 (0.94, 1.05) | 0.802 |  | 0.99 (0.93, 1.05) | 0.746 |  | 0.99 (0.93, 1.05) | 0.804 |
| Age | - | - |  | 1.01 (1.00, 1.01) | 0.003 |  | 1.01 (1.00, 1.01) | 0.011 |  | 1.00 (1.00, 1.00) | 0.054 |
| Ethnicity |  |  |  |  |  |  |  |  |  |  |  |
| White | - | - |  | 1.00 (Reference) |  |  | 1.00 (Reference) |  |  | 1.00 (Reference) |  |
| Asian or Asian British | - | - |  | 1.32 (1.10, 1.58) | 0.002 |  | 1.23 (1.01, 1.49) | 0.041 |  | 1.24 (1.02, 1.51) | 0.031 |
| Black or Black British | - | - |  | 1.88 (1.53, 2.30) | <0.001 |  | 1.82 (1.48, 2.25) | <0.001 |  | 1.81 (1.47, 2.24) | <.0001 |
| Other ethnic group | - | - |  | 1.13 (1.00, 1.28) | 0.052 |  | 1.12 (0.98, 1.27) | 0.090 |  | 1.12 (0.98, 1.27) | 0.088 |
| Education |  |  |  |  |  |  |  |  |  |  |  |
| College or university degree | - | - |  | 1.00 (Reference) |  |  | 1.00 (Reference) |  |  | 1.00 (Reference) |  |
| A/AS level or equivalent | - | - |  | 1.07 (0.98, 1.18) | 0.143 |  | 1.07 (0.98, 1.18) | 0.135 |  | 1.08 (0.98, 119) | 0.114 |
| O levels/GCSEs or equivalent | - | - |  | 1.09 (1.01, 1.17) | 0.023 |  | 1.08 (1.00, 1.16) | 0.038 |  | 1.08 (1.00, 1.16) | 0.050 |
| Other | - | - |  | 0.90 (0.84, 0.96) | 0.002 |  | 0.90 (0.84, 0.97) | 0.004 |  | 0.90 (0.84, 0.96) | 0.003 |
| BMI (kg/m^2^) | - | - |  | 1.00 (0.99, 1.00) | 0.996 |  | 0.90 (0.84, 0.97) | 0.004 |  | 1.00 (0.99, 1.00) | 0.092 |
| Smoking |  |  |  |  |  |  |  |  |  |  |  |
| Never | - | - |  | - | - |  | 1.00 (Reference) |  |  | 1.00 (Reference) |  |
| Previous | - | - |  | - | - |  | 1.04 (0.98, 1.10) | 0.176 |  | 1.04 (0.98, 1.10) | 0.233 |
| Current | - | - |  | - | - |  | 0.91 (0.84, 0.99) | 0.031 |  | 0.92 (0.84, 1.00) | 0.039 |
| Alcohol consumption |  |  |  |  |  |  |  |  |  |  |  |
| Never | - | - |  | - | - |  | 1.00 (Reference) |  |  | 1.00 (Reference) |  |
| Previous | - | - |  | - | - |  | 0.92 (0.80, 1.06) | 0.239 |  | 0.91 (0.79, 1.05) | 0.189 |
| Current | - | - |  | - | - |  | 0.96 (0.86, 1.06) | 0.407 |  | 0.9 (0.86, 1.06)5 | 0.375 |
| [Physical activity](javascript:;) (minutes/week) | - | - |  | - | - |  | 1.00 (1.00, 1.00) | 0.817 |  | 1.00 (1.00, 1.00) | 0.878 |
| Vegetable (tablespoons/day) | - | - |  | - | - |  | 1.01 (1.00, 1.02) | 0.017 |  | 1.01 (1.00, 1.02) | 0.027 |
| Fruit (pieces/day) | - | - |  | - | - |  | 1.00 (0.99, 1.01) | 0.96 |  | 1.00 (0.99, 1.01) | 0.980 |
| [Sugared beverages](javascript:;) |  |  |  |  |  |  |  |  |  |  |  |
| No | - | - |  | - | - |  | 1.00 (Reference) |  |  | 1.00 (Reference) |  |
| Yes | - | - |  | - | - |  | 0.98 (0.92, 1.04) | 0.49 |  | 0.98 (0.92, 1.05) | 0.523 |
| Oily fish |  |  |  |  |  |  |  |  |  |  |  |
| Less than once per week | - | - |  | - | - |  | 1.00 (Reference) |  |  | 1.00 (Reference) |  |
| Once per week | - | - |  | - | - |  | 0.95 (0.90, 1.01) | 0.104 |  | 0.95 (0.90, 1.01) | 0.080 |
| More than once per week | - | - |  | - | - |  | 0.96 (0.89, 1.03) | 0.234 |  | 0.95 (0.89, 1.02) | 0.191 |
| Processed meat |  |  |  |  |  |  |  |  |  |  |  |
| Less than once per week | - | - |  | - | - |  | 1.00 (Reference) |  |  | 1.00 (Reference) |  |
| Once per week | - | - |  | - | - |  | 1.07 (1.00, 1.14) | 0.043 |  | 1.06 (1.00, 1.13) | 0.051 |
| More than once per week | - | - |  | - | - |  | 1.06 (0.99, 1.13) | 0.103 |  | 1.06 (0.99, 1.13) | 0.116 |
| Beef |  |  |  |  |  |  |  |  |  |  |  |
| Less than once per week | - | - |  | - | - |  | 1.00 (Reference) |  |  | 1.00 (Reference) |  |
| Once per week | - | - |  | - | - |  | 0.91 (0.86, 0.97) | 0.002 |  | 0.91 (0.85, 0.97) | 0.002 |
| More than once per week | - | - |  | - | - |  | 0.78 (0.72, 1.12) | <.0001 |  | 0.78 (0.72, 0.85) | <.0001 |
| [Mutton](javascript:;) |  |  |  |  |  |  |  |  |  |  |  |
| Less than once per week | - | - |  | - | - |  | 1.00 (Reference) |  |  | 1.00 (Reference) |  |
| Once per week | - | - |  | - | - |  | 0.98 (0.91, 1.04) | 0.463 |  | 0.98 (0.91, 1.05) | 0.525 |
| More than once per week | - | - |  | - | - |  | 0.96 (0.83, 1.12) | 0.622 |  | 0.97 (0.83,.112) | 0.664 |
| Pork |  |  |  |  |  |  |  |  |  |  |  |
| Less than once per week | - | - |  | - | - |  | 1.00 (Reference) |  |  | 1.00 (Reference) |  |
| Once per week | - | - |  | - | - |  | 1.03 (0.96, 1.10) | 0.370 |  | 1.03 (0.96, 1.10) | 0.388 |
| More than once per week | - | - |  | - | - |  | 1.16 (1.00, 1.34) | 0.049 |  | 1.15 (1.00, 1.33) | 0.058 |
| [FBG](javascript:;) (mmol/L) | - | - |  | - | - |  | - | - |  | 1.14 (1.08, 1.19) | <.0001 |
| [Family history of diabetes](javascript:;) | - | - |  | - | - |  | - | - |  | 1.01 (0.95, 1.07) | 0.831 |

BMI, body mass index; FBG, baseline fasting blood glucose.

Model 0 was analyzed with [single-factor analysis](javascript:;).

Model 1 was adjusted for age, sex, ethnicity, education, and BMI.

Model 2 was adjusted for age, sex, ethnicity, education, BMI, smoking, alcohol consumption, physical activity, and diet (including intake of sugar or sugar-sweetened beverages, vegetables, fruits, processed meats, red meat, and oily fish).

Model 3 was adjusted for age, sex, ethnicity, education, BMI, smoking, alcohol consumption, physical activity, diet, [family history of diabetes](javascript:;), and FBG.

**Table S2.** Baseline characteristics of patients with rheumatoid arthritis

|  | **No hydroxychloroquine**  **(N = 5784)** | **Hydroxychloroquine**  **(N = 383)** | **Overall**  **(N = 6167)** | ***P*** |
| --- | --- | --- | --- | --- |
| **Male, n (%)** | 1791 (30.96) | 77 (20.10) | 1868 (30.29) | <0.001 |
| Age | 61.00 (54.00, 65.00) | 60.00 (54.00, 65.00) | 61.00 (54.00, 65.00) | 0.171 |
| Ethnicity |  |  |  | 0.256 |
| White | 5351 (90.86) | 348 (90.86) | 5699 (92.41) |  |
| Asian or Asian British | 129 (2.23) | 7 (1.83) | 136 (2.21) |  |
| Black or Black British | 75 (1.30) | 5 (1.31) | 80 (1.30) |  |
| Other ethnic group | 229 (90.87) | 23 (6.01) | 252 (4.09) |  |
| Educational level |  |  |  | 0.041 |
| College or university degree | 1240 (21.44) | 98 (25.59) | 1338 (21.70) |  |
| A/AS level or equivalent | 574 (9.92) | 48 (12.53) | 622 (10.09) |  |
| O levels/GCSEs or equivalent | 1470 (25.41) | 94 (24.54) | 1564 (25.36) |  |
| Other | 2500 (43.22) | 143 (37.34) | 2643 (42.86) |  |
| BMI (kg/m^2^) | 27.40 (24.46, 30.84) | 27.11 (23.99, 31.20) | 27.40 (24.43, 30.87) | 0.715 |
| Smoking |  |  |  | 0.023 |
| Never | 2748 (47.94) | 183 (47.91) | 2931 (47.94) |  |
| Previous | 2247 (39.20) | 167 (43.72) | 2414 (39.48) |  |
| Current | 737 (12.86) | 32 (4.16) | 769 (12.58) |  |
| Alcohol consumption |  |  |  | 0.169 |
| Never | 411 (7.13) | 25 (6.53) | 436 (7.09) |  |
| Previous | 423 (7.33) | 38 (9.92) | 461 (7.49) |  |
| Current | 4934 (85.54) | 320 (83.55) | 5254 (85.42) |  |
| [Physical activity](javascript:;) (MET-minutes/week) | 2372.86 (792.00, 2464.00) | 1575.00 (586.00, 2372.86) | 2372.86 (766.50, 2400.00) | <0.001 |
| Vegetables (tablespoons/day) | 5.00 (3.00, 6.00) | 4.00 (3.00, 6.00) | 5.00 (3.00, 6.00) | 0.160 |
| Fruit (pieces/day) | 3.00 (2.00, 4.00) | 3.00 (2.00, 4.00) | 3.00 (2.00, 4.00) | 0.003 |
| [Sugared beverages](javascript:;) |  |  |  | 0.813 |
| No | 1194 (20.64) | 81 (21.15) | 1275 (20.67) |  |
| Yes | 4590 (79.36) | 302 (78.85) | 4892 (79.33) |  |
| Oily fish |  |  |  | 0.059 |
| Less than once per week | 2535 (44.26) | 177 (46.46) | 2712 (44.40) |  |
| Once per week | 2083 (36.37) | 149 (39.11) | 2232 (36.54) |  |
| More than once per week | 1109 (19.36) | 55 (14.44) | 1164 (19.06) |  |
| Processed meat |  |  |  | 0.034 |
| Less than once per week | 2405 (41.87) | 183 (47.91) | 2588 (42.25) |  |
| Once per week | 1709 (29.75) | 111 (29.06) | 1820 (29.71) |  |
| More than once per week | 1630 (28.38) | 88 (23.04) | 1718 (28.04) |  |
| Beef |  |  |  | 0.750 |
| Less than once per week | 3204 (55.87) | 221 (57.85) | 3425 (55.99) |  |
| Once per week | 1788 (31.18) | 114 (29.84) | 1902 (31.09) |  |
| More than once per week | 743 (12.96) | 47 (12.30) | 790 (12.91) |  |
| [Mutton](javascript:;) |  |  |  | 0.087 |
| Less than once per week | 4171 (72.84) | 292 (76.64) | 4463 (73.08) |  |
| Once per week | 1351 (23.59) | 72 (18.90) | 1423 (23.30) |  |
| More than once per week | 204 (3.56) | 17 (4.46) | 221 (3.62) |  |
| Pork |  |  |  | 0.981 |
| Less than once per week | 4242 (74.11) | 280 (73.68) | 4522 (74.08) |  |
| Once per week | 1278 (22.33) | 86 (22.63) | 1364 (22.35) |  |
| More than once per week | 204 (3.56) | 14 (3.68) | 218 (3.57) |  |
| [FBG](javascript:;) (mmol/L) | 4.97 (4.65, 5.23) | 4.94 (4.56, 5.18) | 4.97 (4.65, 5.22) | 0.014 |
| [Family history of diabetes](javascript:;) | 1303 (22.53) | 73 (19.06) | 1376 (22.31) | 0.114 |

**Table S3.** Subgroup and sensitivity analyses of hydroxychloroquine use and diabetes risk in rheumatoid arthritis

|  | **Number of cases/total number (incidence, %)** | |  | **HR (95%CI)** | | ***P*** |
| --- | --- | --- | --- | --- | --- | --- |
|  | **No hydroxychloroquine** | **Hydroxychloroquine** |  | **No hydroxychloroquine** | **Hydroxychloroquine** |  |
| **Overall** | 477/5784 (8.52) | 25/383 (6.53) |  | 1.00 (Ref) | 0.84 (0.75, 0.94) | 0.002 |
| **Subgroup analysis** |  |  |  |  |  |  |
| **Sex** |  |  |  |  |  |  |
| Male | 184/1791 (10.27) | 10/77 (12.99) |  | 1.00 (Ref) | 0.74 (0.57, 0.94) | 0.016 |
| Female | 293/3993 (7.34) | 15/306 (4.90) |  | 1.00 (Ref) | 0.88 (0.78, 0.99) | 0.035 |
| **Age** |  |  |  |  |  |  |
| ≤60 years | 228/2867 (7.95) | 16/207 (7.73) |  | 1.00 (Ref) | 0.85 (0.73, 0.99) | 0.038 |
| >60 years | 249/2917 (8.54) | 9/176 (5.11) |  | 1.00 (Ref) | 0.81 (0.69, 0.95) | 0.009 |
| **FBG** |  |  |  |  |  |  |
| <6.1 (mmol/L) | 422/5531 (7.63) | 23/371 (6.20) |  | 1.00 (Ref) | 0.83 (0.74, 0.93) | <0.001 |
| ≥6.1 (mmol/L) | 55/253 (21.74) | 2/12 (16.67) |  | 1.00 (Ref) | 1.30 (0.61, 2.74) | 0.500 |
| **BMI** |  |  |  |  |  |  |
| <25 kg/m^2^ | 50/1703 (2.94) | 2/122 (1.64) |  | 1.00 (Ref) | 0.76 (0.63, 0.92) | 0.005 |
| 25–30kg/m^2^ | 142/2333 (6.09) | 7/143 (4.90) |  | 1.00 (Ref) | 0.86 (0.72, 1.02) | 0.089 |
| ≥30 kg/m^2^ | 285/1748 (16.30) | 16/118 (13.56) |  | 1.00 (Ref) | 0.89 (0.72, 1.09) | 0.266 |
| **Course of disease** |  |  |  |  |  |  |
| <10 years | 244/3092 (7.89) | 15/243 (7.03) |  | 1.00 (Ref) | 0.86 (0.74, 0.99) | 0.038 |
| ≥10 years | 233/2692 (8.66) | 10/170 (0.82) |  | 1.00 (Ref) | 0.82 (0.70, 0.96) | 0.017 |
| **Sensitive analysis** |  |  |  |  |  |  |
| **Ethnicity: White** | 415/5351 (7.76) | 21/348 (6.03) |  |  | 0.80 (0.72, 0.90) | <0.001 |
| **Excluding too short a follow-up** |  |  |  |  |  |  |
| ≤1 year | 458/5765 (7.94) | 22/380 (5.79) |  | 1.00 (Ref) | 0.84 (0.75, 0.94) | 0.002 |
| ≤2 years | 432/5739 (7.53) | 21/379 (5.54) |  | 1.00 (Ref) | 0.84 (0.75, 0.94) | 0.002 |
| **Eliminating poor health** | 469/5725 (8.19) | 25/381 (6.56) |  | 1.00 (Ref) | 0.84 (0.76, 0.94) | 0.002 |
| **Excluding loss to follow-up due to death** | 477/5096 (9.36) | 25/322 (7.76) |  | 1.00 (Ref) | 0.85 (0.76, 0.96) | 0.010 |
